# Quality and Cost Interventions During the Extended Perinatal Period to Increase Family Planning Use in Kinshasa, DRC: Results From an Initial Study

**DOI:** 10.9745/GHSP-D-18-00075

**Published:** 2018-10-03

**Authors:** Leah Jarvis, Jane Wickstrom, Gwyneth Vance, Jewel Gausman

**Affiliations:** a EngenderHealth, New York, NY, USA. Now with Population Council, New York, NY, USA.; b EngenderHealth, New York, NY, USA. Now with Bill & Melinda Gates Foundation, Seattle, WA, USA.; c Independent consultant, New Orleans, LA, USA.; d Harvard T. H. Chan School of Public Health, Amman, Jordan.

## Abstract

**Background:**

Most women worldwide do not desire another pregnancy within a year after giving birth, but uptake of modern contraception during this time period is low. We independently tested 2 approaches to increasing contraceptive uptake and the 2 approaches combined using a quasi-experimental study design in Kinshasa, the Democratic Republic of the Congo.

**Methods:**

The primary analytic data came from client exit interviews conducted post-intervention (N=563) from 4 study groups. The first arm (n=150) received free family planning, and the second arm (n=113) a quality inputs intervention involving systematic screening, referral, and immediate provision of long-acting reversible contraceptives (LARCs) after labor and delivery. The third arm (n=150) received a combination of the 2 interventions, and the fourth (n=150) no intervention. Family planning service statistics were also collected throughout the intervention period.

**Results:**

Women in the quality arm (odds ratio [OR]=4.5; 95% confidence interval [CI], 1.8 to 10.9) and free/quality arm (OR=6.7; 95% CI, 2.8 to 16.1) were more likely to be properly screened for family planning than women in the control group, but paper referral was seldom implemented in any group. Women in the free arm (OR=3.8; 95% CI, 1.6 to 9.0) and in the free/quality arm (OR=11.0; 95% CI, 4.3 to 27.9) were more likely than the control group to report being properly counseled on family planning. Clients were more likely to be modern contraceptive users (excluding condoms) in the free arm (OR=3.2; 95% CI, 1.4 to 7.2) and in the free/quality arm (OR=8.6; 95% CI, 3.9 to 19.0) than in the control group. Clients in all study arms were more likely to use a LARC compared with the control group (Quality arm: OR=2.9; 95% CI, 1.1 to 7.9. Free arm: OR=5.6; 95% CI, 2.3 to 13.7. Free/quality arm: OR=8.4; 95% CI, 3.4 to 20.6). Service statistics from the combined intervention arm showed that a significantly greater proportion of family planning adoption occurred within the immediate postpartum period (0 to 2 days) in the quality arm (*P*<.001) and free/quality arm (*P*<.001) than in the control arm. Quality inputs, free contraceptives, and the combined intervention had positive impacts on aspects of screening and contraceptive uptake. The combined intervention performed best by all measures.

**Conclusion:**

Providing family planning, including LARCs, in the immediate postpartum period, implementing a systematic screening and referral system, and providing free methods may improve family planning access and uptake in the extended perinatal period in this environment.

## Abstract (precis)

The combined intervention of free contraceptives plus a set of quality inputs for family planning during the extended perinatal period, including provision of long-acting methods immediately postpartum, had the strongest effect on use of modern contraceptives, especially long-acting methods.

Résumé en français à la fin de l'article.

## INTRODUCTION

An estimated 95% of postpartum women worldwide do not desire another pregnancy within 12 months of giving birth; however, due to low uptake of modern family planning methods, the risk of unplanned pregnancies in this group remains high.^1^^–^^4^ In a 12-country analysis, nearly three-quarters of postpartum women in sub-Saharan Africa were estimated to have an unmet need for family planning,^4^ an estimate that has remained fairly consistent over the past decade.^2^

Barriers to reducing unmet need for postpartum family planning (PPFP) range from sociocultural and informational^5^^–^^11^ to structural and economic. Data show that family planning cost alone, particularly for long-acting reversible contraceptives (LARCs)—i.e., intrauterine devices (IUDs) and hormonal implants—and permanent methods—i.e., male and female sterilization—can be a substantial barrier to uptake.^11^^–^^14^ These methods often entail higher up-front costs than do short-acting methods (i.e., condoms, oral contraceptives, and injectables).^15^ Although LARCs and permanent methods may be well suited to the reproductive intentions of postpartum women, previous research indicates that when postpartum women do use a method, they overwhelmingly use short-acting methods.^2^^,^^16^

Strategies such as voucher systems have been used to address economic barriers to family planning; however, specific data are limited on how providing free contraceptives may impact PPFP use^17^^–^^19^ and on how cost and integration interventions may work together to increase PPFP use. Integration generally refers to approaches to remediation of PPFP barriers within the health system. These efforts tend to focus on the extended perinatal period (EPP)—defined as pregnancy through the first year postpartum—visit structure, whereby exposure to antenatal care (ANC), labor and delivery (L&D), postnatal care (PNC), and infant/child health and immunization services ensure that women have frequent, scheduled contact with the health system. World Health Organization (WHO) and other international guidelines recommend using these health system contact points to provide family planning information and services and expand the availability of the range of family planning methods available. Provider training on LARCs and permanent methods delivery is an important component of the expansion.^20^^,^^21^

Much research on PPFP use to date, however, has been focused on integrating family planning messages and referrals into a single delivery point, such as immunization or ANC services, as opposed to providing services through many delivery points in a health facility. The results have been mixed.^6^^,^^7^^,^^22^^–^^25^ Research in Rwanda and Liberia demonstrated increased family planning uptake by providing referrals for co-located family planning services to mothers bringing their children to immunization services.^7^^,^^24^ In contrast, research on integration of family planning messages into immunization services in Ghana and Zambia found no effect on family planning uptake.^25^ In other contexts, research has demonstrated that the provision of family planning counseling and services during labor and delivery and postpartum care can increase uptake of family planning immediately postpartum; this is now considered a “proven” high impact practice.^10^^,^^21^

Provision of family planning counseling and services during labor and delivery can increase contraceptive uptake immediately postpartum.

### The Democratic Republic of the Congo Context

In the Democratic Republic of the Congo (DRC), the national modern contraceptive prevalence rate among married women of reproductive age is extremely low (7.8%), while unmet need is high (27.7%) and only one-third of facilities offer any family planning services.^26^^,^^27^ The Ministry of Health (MOH) is working to address these issues by making a national commitment as part of the Family Planning 2020 (FP2020) global movement and establishing a country action plan to increase PPFP access.^28^^–^^31^ EngenderHealth's Expand Family Planning Project (ExpandFP) (2013–2020) is one of many efforts funded by the Bill & Melinda Gates Foundation and other donors to support the DRC MOH to move toward their FP2020 and PPFP goals.

In the DRC, ExpandFP focused efforts in Kinshasa, where unmet need is relatively high (22.6%) among married women of reproductive age and facility-based delivery is nearly universal.^26^^,^^32^ This environment presented opportunities to reach women at health facilities who wanted and needed family planning during the EPP. However, throughout the DRC, family planning is not routinely provided for free in either the public or the private sector. The median cost to the client for short- and long-acting methods ranges from less than US$1 for oral contraceptives to more than US$10 for an implant, with variation among facilities.^15^ In a country where the per capita gross national income is about US$460 per year, contraceptive costs may pose an important barrier to family planning use.^33^ Therefore, we wanted to understand how eliminating cost as a barrier could affect PPFP uptake.

The cost of contraception in the DRC can be a barrier to family planning use.

To assist the DRC MOH National Reproductive Health Program, ExpandFP conducted an operational study to determine how to best increase family planning uptake in the EPP. Two interventions were evaluated separately and in combination with each another. The first intervention provided free contraceptives. The second, referred to as “quality inputs,” focused on the perinatal contacts within the health system at all service delivery points in the EPP; these include systematic screening and referral in child health/immunization, ANC, and PNC services, along with additional training on family planning counseling and immediate provision of LARCs in L&D wards. We hypothesized that both the free contraceptives and quality inputs interventions would increase uptake in the EPP, but that the combined intervention would have the greatest effect.

## METHODS

**FIGURE 1:**
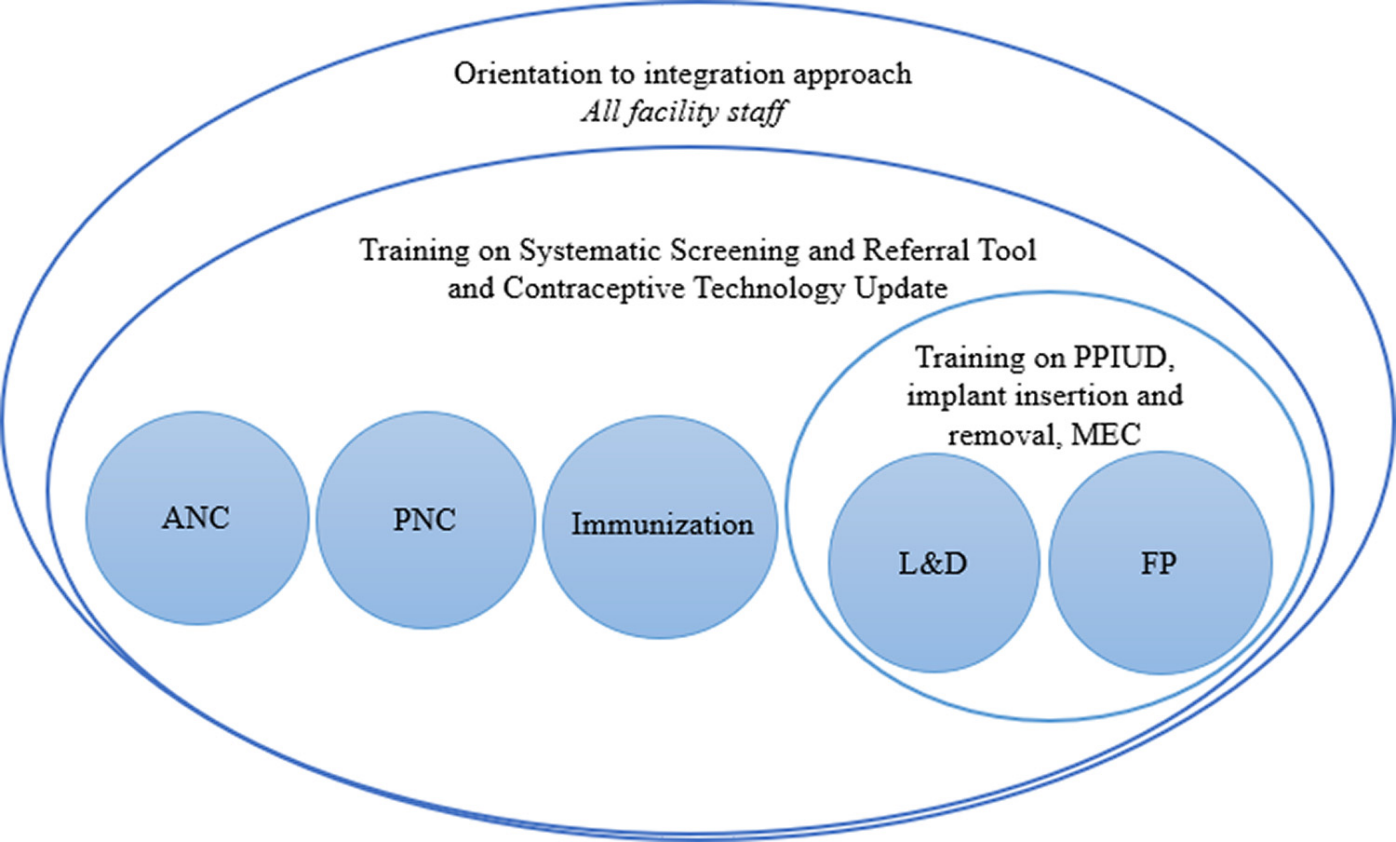
Whole-Site Training Model Abbreviations: ANC, antenatal care; FP, family planning; L&D, labor and delivery; MEC, medical eligibility criteria; PNC, postnatal care; PPIUD, postpartum insertion of an intrauterine device.

The research used a 4-group, nonrandomized, posttest study design. Data evaluating the primary outcomes were collected from client exit interviews and were bolstered by family planning service statistics. To be eligible for inclusion in the study, facilities in Kinshasa were required to meet the following minimum criteria: (1) have no other implementing partners; (2) have a relatively high monthly caseload of L&D patients, with an average of at least 30 per month; and (3) serve a peri-urban population. The 4 facilities (2 hospitals and 2 maternity referral centers) that met these criteria and were willing to participate were purposively chosen under advisement from the MOH and assigned to 1 of 4 study arms (Figure 1). The Arm 1 (“quality”) facility was assigned to the quality inputs intervention, the Arm 2 (“free”) facility was assigned to the free contraceptives intervention, the Arm 3 (“free/quality”) facility was assigned to the free contraceptive and quality inputs intervention, and the Arm 4 facility served as the control.

Study activities were initiated in February 2016 and concluded in June 2017. Initiation of the interventions was staggered over 3 months across the 3 treatment sites and lasted a total of 12 months at each facility.

### Interventions

#### Quality Inputs (Arms 1 and 3)

The quality inputs intervention consisted of 3 components: (1) clinical training and provision of equipment for postpartum insertion of the IUD (PPIUD), (2) training on WHO's *Medical Eligibility Criteria (MEC) for Contraceptive Use*,^34^ and (3) introduction of a systematic screening and referral tool for family planning.

A whole-site training approach was used to deliver the training and education components of the quality inputs intervention.

These components were launched through whole-site training: a facility-wide approach intended to meet the learning needs of all staff in various departments and to encourage teamwork among them.^35^^–^^38^ The whole-site training consisted of a 7-day, facility-based training for providers and non-clinicians alike. Providers and facility staff participated in relevant parts of the training according to their role in the intervention (Figure 1). ANC, PNC, and immunization providers were expected to screen clients for family planning need, current or future, and refer the clients as needed. L&D providers were expected to do the same, but also had the ability to counsel on and deliver some methods within their department. Family planning providers were not expected to screen and refer, but rather to counsel and deliver family planning methods.

The trainers and facility supervisors first oriented staff from the entire facility over a 2-day period to the integration approach and the goals of the intervention, at the same time assessing training and equipment needs. Then, providers were divided into subgroups for specialized training according to their primary function in the facility. Over 5 days, providers from the family planning and L&D departments were trained on postpartum insertion of an IUD, provided with needed PPIUD equipment, and refreshed on implant insertion and removal. The clinical training also included an update on MEC for postpartum women and a review of rights-based family planning counseling.^39^ Trainees were assessed for competency using anatomical models, and supervised PPIUD insertions were conducted with available clients for on-the-job coaching on the last day of the training. As a result of the training, providers were able to provide PPIUDs, implants, and other methods within the L&D department immediately postpartum.

Providers from departments within the facility that women frequent throughout the EPP—specifically, ANC, L&D, PNC, and child health/immunization—were given a contraceptive technology update and trained on the use of the screening and referral tool.

The adapted paper-based systematic screening and referral tool, developed by the Population Council and IntraHealth International and tested in several resource-challenged settings,^40^^–^^43^ was designed to help providers screen women for family planning need and provide written referrals. The first page of the tool is an abbreviated assessment of the client's reproductive intentions, pregnancy and delivery history, family planning practices and satisfaction, and desire for additional family planning information or services. When a client indicates interest in additional information or services, the provider is prompted to continue to a written referral form, which is then sent with the client to the family planning unit, where family planning counseling is continued and a method is provided if desired. Both screening forms and referral forms were filed and used as a study monitoring mechanism throughout the implementation period.

The study adapted a paper-based systematic screening and referral tool designed to help providers screen women for family planning need and provide written referrals.

#### Free Contraceptives (Arms 2 and 3)

To address economic barriers to family planning access, all contraceptives were provided free of charge in Arms 2 and 3 in both the L&D and family planning units. Prior to the study, the 4 study sites offered a range of family planning methods for a fee, with the exception of male condoms, which were free at all 4 facilities. Costs to clients at these facilities varied from US$0.30 to $10 for short-acting methods and from US$5 to $10 for LARCs. Female sterilization cost US$100 to $250 at 3 of the facilities, but was only available as part of a cesarean delivery. ExpandFP provided a monthly stipend to the facilities providing free contraception that was approximately equal to lost revenue from charging for family planning; the amount of this stipend was negotiated prior to study launch. No targeted demand generation activities were conducted in any study site, as the intervention was intended to be facility-based only.

Monthly stipends were provided to facilities to compensate them for providing free contraception to clients during the study period.

#### Method Availability (Arms 1, 2, 3, and 4)

In all 4 facilities, clients could access IUDs, implants, injectables, oral contraceptives, and male condoms in the family planning department throughout the duration of the intervention, and facilities were supported to ensure no stock-outs occurred. Clients could not access family planning methods in ANC, PNC, or immunization departments in any of the 4 facilities.

In the quality intervention sites only (Arms 1 and 3), clients could obtain IUDs, implants, or oral contraceptives in L&D in the immediate postpartum period. They could also be referred for family planning if they chose not to adopt family planning at that time but were interested in future use.

Female sterilization was unavailable in the quality arm facility. Vasectomy was not available at any of the facilities. The project did not aim to increase the availability of permanent methods.

### Outcomes of Interest

The main study outcomes of interest related to family planning service provision quality and family planning uptake. Outcomes were assessed in the service delivery departments or study populations to which they were applicable (Table 1). Secondarily, we assessed how well the approaches tested targeted women in the EPP, and how the approaches affected method mix among family planning clients.

**TABLE 1.** Primary Study Outcomes, Criteria, and Analysis Set

Outcome (Yes/No) by Source of Data	Criteria	Analysis Set
**Client Exit Interview**		
*Quality FP Service Delivery*		
Properly screened for FP	Client reported that (1) her provider either asked if she wanted more children or when she wanted more children and (2) provider asked client if she was interested in FP.	Women interviewed at ANC, PNC, immunization, and L&D services
Properly referred for FP	Client reported that (1) her provider asked if she was interested in FP, (2) she told the provider she was interested, and (3) the interviewer was able to observe the paper referral slip in client's hand.	Women interviewed at ANC, PNC, and immunization services
Properly counseled on FP	Client reported that her provider (1) gave her a chance to ask questions; (2) if so, the answers to questions were satisfactory; (3) the provider discussed advantages of methods; (4) the provider discussed side effects; and (5) the provider told client what to do if she experienced side effects.	Women interviewed at L&D and FP services
Properly counseled on LARC	Client reported that her provider told her (1) where her LARC could be removed; (2) when her LARC should be removed, based on maximum duration of use; and (3) that she could have her LARC removed at any time.	LARC adopters
*FP Method Use*		
Modern FP user	Client reported that she was using one of the following FP methods or that she had received one of the following methods on the day of interview: *male/female sterilization, IUD, implant, oral contraceptives, male/female condoms, emergency contraception, standard days method, or lactational amenorrhea method.*	All nonpregnant women
Modern non-condom FP user	Same as previous, except male/female condoms were excluded from the list of methods.	All nonpregnant women
LARC user	Same as previous, except only includes users of IUDs or implants.	All nonpregnant women
**Service Statistics**		
*Postpartum Distribution of FP Clients*		
FP client in the EPP	Client received a modern FP method and was within 12 months of last delivery.	All FP clients
FP client in immediate postpartum period	Client received a modern FP method and was within 2 days of last delivery.	All FP clients

Abbreviations: ANC, antenatal care; EPP, extended perinatal period; FP, family planning; IUD, intrauterine device; L&D, labor and delivery; LARC, long-acting reversible contraceptive; PNC, postnatal care.

Family planning service provision quality and family planning uptake were the primary study outcomes of interest.

To evaluate the quality domain, binary outcomes for family planning screening, family planning referral, family planning counseling, and LARC counseling were assessed; criteria for each outcome are described in Table 1. From client exit interview data, affirmative answers to all criteria were necessary to justify a “Yes” classification for each outcome. The analysis set (i.e., denominator data) used for each of the outcomes varied based on the type of service delivery point. For example, family planning providers were not expected to screen for family planning need or refer for family planning, since a client presenting had already expressed interest in family planning by coming to that department, so screening and referral were not evaluated among clients exiting family planning service departments.

Evaluation of the family planning uptake domain was structured around clients' use of modern methods among all nonpregnant women enrolled in the study, either including or excluding condoms (Table 1). Condoms were excluded from some analyses to look specifically at female-controlled methods used exclusively for pregnancy prevention and to better isolate the impact of free contraception, since condoms were free in all facilities.

A subanalysis compared aspects of counseling and method provision in L&D to family planning wards to help isolate how the intervention affected these service delivery points specifically and identify areas for improvement.

To evaluate the extent to which the intervention reached women in the EPP, service statistics were examined to determine the proportion of all family planning clients who were within 0 to 2 days, 3 days to 6 weeks, and more than 6 weeks to 12 months postpartum. They were also used to compare the method mix by study arm.

### Data Collection

The primary sources of data for the study were (1) structured client exit interviews from L&D, family planning, ANC, PNC, and child immunization/health departments and (2) routine family planning service statistics collected throughout the intervention.

For client exit interviews, women were eligible for participation if they were between 18 and 49 years of age. Clients provided written consent and were interviewed in their preferred language (French or Lingala). Client interviews were used to collect data on client sociodemographic characteristics and reproductive history and intentions as well as experiences with family planning screening and referral, the content of family planning counseling, and family planning use/adoption from the delivery point they had attended on the day of the interview. Questions also gauged client perspectives on the cost of methods and how cost affected method selection. Although clients who received more than 1 service in a day were not interviewed multiple times, it is possible that a client who returned to the facility during the weeks of data collection could have been interviewed more than once.

The interview forms were field-tested in both French and Lingala and were administered over a 5-week period in participants' preferred language by trained data collectors. Interviews were conducted from March to April 2017, after the intervention had been implemented for 9 to 12 months in each facility, depending on the date of initiation.

Family planning service statistics were extracted from the family planning and L&D registers for 12 months following the introduction of the intervention in each facility. A column for “date of last delivery” was added to the registers, and the registers were reviewed routinely by project staff to ensure completeness and accuracy. Data obtained from service statistics included family planning method received, date of service, and date of last delivery, allowing for calculation of the postpartum period.

### Sampling

Sample size was calculated to be able to detect a 15 percentage point difference in the percentage of clients screened for family planning in intervention groups compared with the control. Assuming a 25% level of screening in the control group, and using a 2-sided test with an alpha of 0.05 and 80% power, we estimated that 100 clients per facility were needed, split evenly across ANC, PNC, immunization, and L&D services, where screening was expected to occur.

**FIGURE 2:**
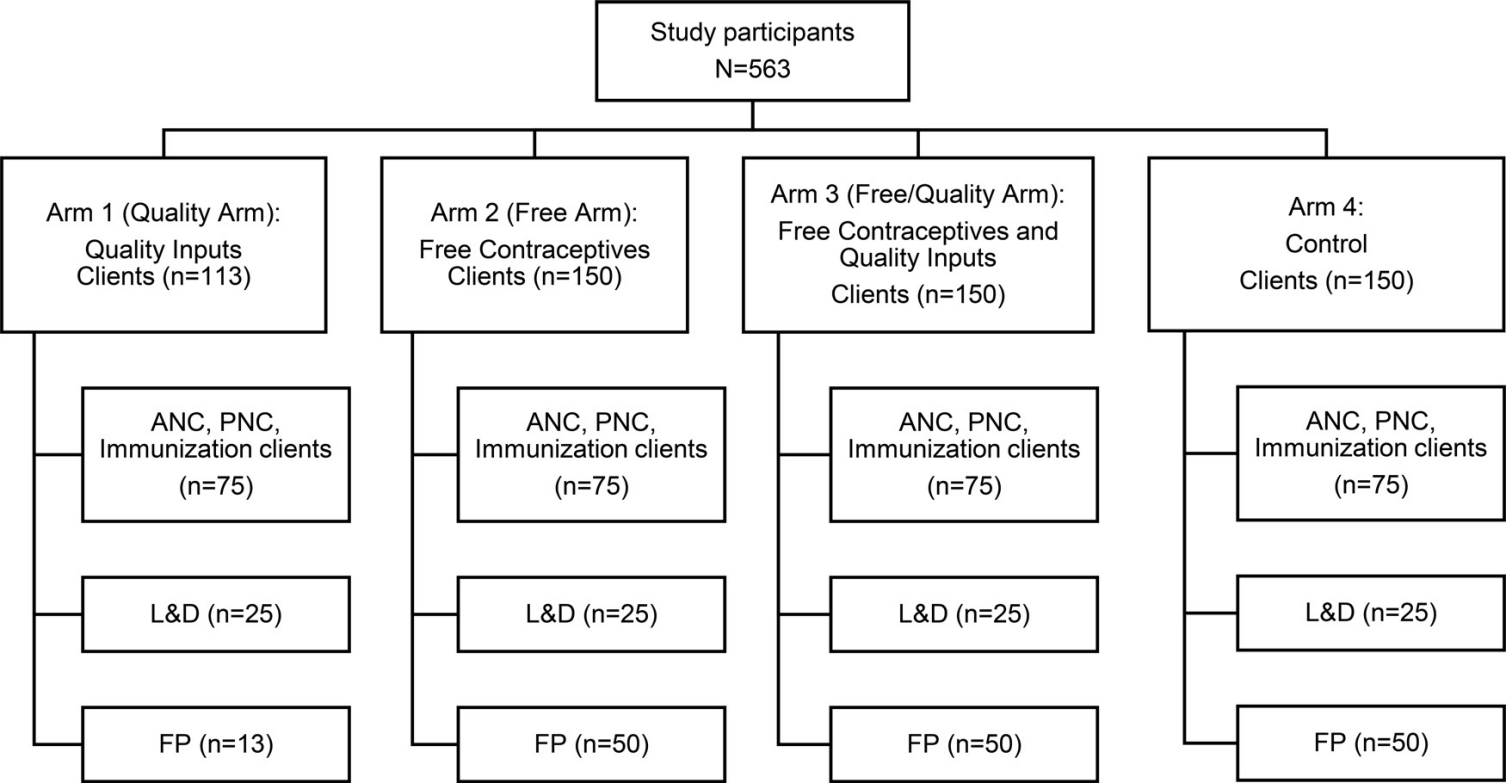
Study Participants, by Study Arm and Facility Department Abbreviations: ANC, antenatal care; FP, family planning; L&D, labor and delivery; PNC, postnatal care.

Because we also sought to assess outcomes related to family planning counseling and service provision and overcome a low family planning client load in the control arm, we interviewed an independent sample of 50 women in the family planning unit in each facility. In total, 25 clients were sampled for a quantitative interview in each of the PNC, ANC, L&D, and immunization units, and 50 clients in family planning, resulting in about 150 participants per facility. All clients meeting the study criteria were asked to participate in an interview; recruiting continued until the sample size targets were reached (Figure 2).

### Data Analysis and Statistical Tests

Client interview data were double-entered into Epi Info (U.S. Centers for Disease Control and Prevention, Atlanta, GA, USA) and analyzed in Stata version 14 (StataCorp, College Station, TX, USA). The main outcomes were evaluated through the development of logistic regression models (Table 1). Dummy variables representing study groups were included as covariates in the crude models; odds ratios (ORs) and 95% confidence intervals (CIs) were reported. The final models used for comparison, however, were adjusted for potentially confounding factors. To determine the factors to include as covariates in adjusted models, client background characteristics that might also relate to family planning uptake were assessed. This included client age, parity, marital status, education level, occupation, religion, ever-use of family planning methods, ever-use of LARCs, pregnancy status, postpartum period, fertility intentions, and socioeconomic status (SES). An indicator of SES was derived from several questions on assets, income, and savings through principal component analysis (PCA).^44^ Briefly, PCA was used to reduce the set of SES indicators to an index score with a mean of 0 and a standard deviation (SD) of 1. SES-related variables used to construct the score were based on yes/no responses to questions about possessions, cash savings, land ownership, assets that could be used to generate income, and income earned outside the home. A PCA value of 0.32, for example, meant that the women in this group were above the mean SES of all women in the study.

Client background characteristics were used to determine what factors might contribute to family planning uptake.

Differences between each study group and the control on these factors were tested using chi-square tests for proportions and *t* tests for means. If *P*≤.05 at the 95% CI for any client characteristics, they were treated as potential confounders and included in the adjusted logistic models.

Family planning service statistics were analyzed by calculating the proportions of family planning clients who chose each method and the proportions who fell within the EPP. The postpartum period was further disaggregated to the immediate postpartum (0 to 2 days), the standard postpartum (0 to 6 weeks), and the extended postpartum (0 to 12 months) periods. Nulliparous women and women for whom the date of last delivery was unknown were excluded. The proportions of women in each category were compared between study arms and the control arm, using chi-square tests. Receiving a method within 2 days of delivery indicated that the method had been provided within the L&D department. Analyzing change over time in proportion of family planning clients in the EPP was not possible due to the unavailability of baseline data.

### Ethical Approval

The protocol and tools were approved by the Western International Review Board in the United States and by the University of Kinshasa School of Public Health Ethical Review Committee.

## RESULTS

### Client Characteristics

Data were collected from a total of 563 clients (Figure 2); low client flow in Arm 1 resulted in a lower than desired sample size. Women in all 3 intervention groups were similar to the control group in age, marital and pregnancy status, desire for children in the next 2 years, and ever-use of a LARC (Table 2). Women in all 3 intervention groups had significantly higher mean pregnancies compared with the control group. Women in the free contraceptives groups (Arms 2 and 3) had significantly lower mean SES PCA scores, educational attainment, and a higher mean number of children than the control group. The free contraceptives-only group (Arm 2) also had fewer respondents within 12 months postpartum and a lower proportion of participants who ever used modern family planning. Other covariates found to be significantly different in at least 1 arm compared with the control arm were religious affiliation and working outside the home.

**TABLE 2.** Sociodemographic Characteristics of Clients, by Study Arm

	Quality Arm 1 (n=113)	Free Arm 2 (n=150)	Free/Quality Arm 3 (n=150)	Control Arm 4 (n=150)
Age, years, mean (SD)	29.2 (5.7)	29.2 (6.2)	28.1 (6.2)	27.9 (5.9)
No. of pregnancies/woman, mean (SD)	3.6 (1.9)**	4.0 (2.2)**	3.5 (1.8)**	2.8 (1.7)
No. of children/woman, mean (SD)	2.6 (1.6)	3.3 (2.1)**	3.0 (1.8)**	2.3 (1.5)
SES score, mean (SD)	0.58 (0.9)*	−0.26 (0.9)**	−0.49 (1.0)**	0.32 (0.9)
Married or living as married, %	88.5	87.3	87.3	86.7
Education: completed secondary, %	66.4	33.3**	32.0**	68.0
Religion: Christian,^a^ %	72.6*	80.0	76.7*	86.0
Works outside the home, %	41.6*	68.0*	52.0	55.3
Pregnant, %	21.1	18.0	16.7	16.7
Within 12 months postpartum, %	77.7	63.8*	68.7	74.8
Does not want child in next 2 years, %	93.8	94.0	96.7	94.7
Ever used modern family planning, %	69.0	51.3*	60.0	64.7
Ever used LARC, %	9.7	9.3	8.0	6.7

Abbreviations: LARC, long-acting reversible contraceptive; SD, standard deviation; SES, socioeconomic status.

* *P*<.05;

** *P*<.001.

a Christian includes Lutheran, Pentecostal, Protestant, and nondenominational.

### Screening and Referral

The first outcome of interest was the proportion of women attending ANC, PNC, immunization, or L&D departments who were properly screened for family planning need. Women in the quality arm (Arm 1) (OR=4.5; 95% CI, 1.8 to 10.9) and free/quality arm (Arm 3) (OR=6.7; 95% CI, 2.8 to 16.1) were significantly more likely to have been properly screened for family planning than were women in the control group. In the free/quality arm, approximately one-third (n=36, 36.0%) of women reported having been properly screened, compared with one-quarter (n=26, 26%) of women in the quality arm and approximately one-tenth in the free arm (n=11, 11.0%) and the control group (n=8, 8.0%) (Table 3).

**TABLE 3.** Crude and Adjusted Odds Ratios for Proper Family Planning Screening, Referral, and Counseling

Study Arm	Proper Family Planning Screening^a^ (n=400)				Proper Family Planning Referral^b^ (n=300)				Proper Family Planning Counseling^c^ (n=263)			
	No.	Positive Response No. (%)	Crude OR (95% CI)	Adjusted^d^ OR (95% CI)	No.	Positive Response No. (%)	Crude OR (95% CI)	Adjusted^d^ OR (95% CI)	No.	Positive ResponseNo. (%)	Crude OR (95% CI)	Adjusted d OR (95% CI)
1: Quality	100	26 (26.0)	4.0 (1.7, 9.5)*	4.5 (1.8, 10.9)*	75	3 (4.0)	NA	NA	38	8 (21.1)	1.6 (0.6, 4.3)	1.7 (0.6, 4.8)
2: Free	100	11 (11.0)	1.4 (0.5, 3.7)	1.5 (0.6, 4.0)	75	0 (0.0)	NA	NA	75	32 (42.7)	4.3 (2.0, 9.5)**	3.8 (1.6, 9.0)*
3: Free/quality	100	36 (36.0)	6.5 (2.8, 14.8)**	6.7 (2.8, 16.1)**	75	0 (0.0)	NA	NA	75	46 (61.3)	9.2 (4.2, 20.3)**	11.0 (4.3, 27.9)**
4: Control	100	8 (8.0)	ref	ref	75	0 (0.0)	NA	NA	75	11 (14.7)	ref	ref

Abbreviations: ANC, antenatal care; CI, confidence interval; L&D, labor and delivery; OR, odds ratio; PNC, postnatal care; SES, socioeconomic status.

* *P*<.005;

** *P*<.001.

a ANC, PNC, Immunization, L&D.

b ANC, PNC, Immunization.

c L&D, Family Planning.

d Adjusted for SES, education level, religion, marital status, parity, postpartum status, and ever-use of modern contraception.

The receipt of proper referral was assessed among women obtaining services from the ANC, PNC, and immunization service delivery points. The results indicate that no women in the control, free, or free/quality arms were properly referred; only a few women in the quality arm received a written referral (n=3, 4.0%) (Table 3).

Despite providers receiving training to give appropriate family planning referrals, women were not properly referred, if at all, from ANC, PNC, or immunization delivery points.

### Family Planning Counseling

Proper family planning counseling, as reported by clients, was assessed only among women attending L&D and family planning services, as providers in other services were expected to screen and refer only, not offer comprehensive family planning counseling. Women in the free arm (Arm 2) (OR=3.8; 95% CI, 1.6 to 9.0) and free/quality arm (Arm 3) (OR=11.0; 95% CI, 4.3 to 27.9) were significantly more likely to report receipt of proper family planning counseling compared with those in the control group. No difference was detected in counseling between the quality-only group and control group (Table 3).

### Family Planning Use

The likelihood of being a family planning user was examined among all nonpregnant participants in all departments, including women not in the EPP (Table 4). Women in the free/quality arm were more likely to be a modern family planning user than those in the control group (OR=2.3; 95% CI, 1.2 to 4.3), while those in the quality arm were less likely than those in the control group (OR=0.4; 95% CI, 0.2 to 0.9). When analyses were restricted to modern method use excluding condoms, women in the free arm (OR= 3.2; 95% CI, 1.4 to 7.2) and free/quality arm (OR=8.6; 95% CI, 3.9 to 19.0) were significantly more likely to use modern methods compared with women in the control group. Reported use of LARCs was significantly higher across all intervention groups compared with the control (Quality arm: OR=2.9; 95% CI, 1.1 to 7.9. Free arm: OR=5.6; 95% CI, 2.3 to 13.7. Free/quality arm: OR=8.5; 95% CI, 3.4 to 20.6). The number of IUD users was too small to make statistical comparisons. Clients in both arms with free contraceptives were significantly more likely to be implant users compared to the control (Free arm: OR= 5.7; 95% CI, 2.2 to 14.4. Free/quality arm: OR=5.6; 95% CI, 2.2 to 14.4) (Table 4).

**TABLE 4.** Crude and Adjusted Odds Ratios for Family Planning Use Among All Nonpregnant Women

Study Arm	Modern FP Use^a^ (n=461)			Modern FP Use, Excluding Condoms^a^ (n=461)			LARC Use^a^ (n=461)			IUD Use (n=461)			Implant Use (n=461)		
	Positive Response No. (%)	Crude OR (95% CI)	Adj^b^ OR (95% CI)	Positive Response No. (%)	Crude OR (95% CI)	Adj^b^ OR(95% CI)	Positive Response No. (%)	Crude OR (95% CI)	Adj^b^ OR (95% CI)	Positive Response No. (%)	Crude OR (95% CI)	Adj^b^ OR (95% CI)	Positive Response No. (%)	Crude OR (95% CI)	Adj^b^ OR (95% CI)
1: Quality (n=88)	18 (20.5)	0.4 (0.2, 0.8)*	0.4 (0.2, 0.9)*	14 (15.9)	0.8 (0.4, 1.7)	1.4 (0.6, 3.2)	11 (12.5)	2.1 (0.8, 5.4)	2.9 (1.1, 7.9)*	3 (3.4)	--^c^	--^c^	8 (9.1)	1.7 (0.6, 4.8)	2.3 (0.8, 6.9)
2: Free (n=123)	53 (43.1)	1.2 (0.7, 2.0)	0.9 (0.5, 1.8)	52 (42.3)	3.2 (1.8, 5.8)***	3.2 (1.4, 7.2)**	37 (30.1)	6.3 (2.8, 14.2)***	5.6 (2.3, 13.7)***	1 (0.8)	--^c^	--^c^	36 (29.3)	7.0 (3.0, 16.4)***	5.7 (2.2, 14.4)***
3: Free/quality(n=125)	74 (59.2)	2.3 (1.4, 3.9)**	2.3 (1.2, 4.3)*	72 (57.6)	6.0 (3.4, 10.7)***	8.6 (3.9, 19.0)***	45 (36.0)	8.2 (3.7, 18.4)***	8.4 (3.4, 20.6)***	9 (7.2)	--^c^	--^c^	36 (28.8)	6.8 (2.9, 16.0)***	5.6 (2.2, 14.4)***
4: Control (n=125)	48 (38.4)	ref	ref	23 (18.4)	ref	ref	8 (6.4)	ref	ref	1 (0.8)	ref	ref	7 (5.6)	ref	ref

Abbreviations: Adj, adjusted; CI, confidence interval; FP, family planning; IUD, intrauterine device; LARC, long-acting reversible contraceptive; OR, odds ratio; SES, socioeconomic status.

* *P*<.05;

** *P*<.005;

*** *P*<.001.

a Nonpregnant women.

b Adjusted for SES, education level, religion, marital status, parity, postpartum status, and ever-use of modern contraception.

c Odds ratios not presented due to small cell size.

Women in the free arm were more likely to use modern methods, and the use of LARCs was higher across all intervention groups compared with the control group.

### Subanalysis of Labor and Delivery and Family Planning Uptake and Counseling

Subsets of L&D (n=25) and family planning (n=50) client interview data were analyzed separately (Table 5). In the free/quality arm, 60.0% (n=15) of women in L&D received a method; no women in L&D received a method in any other study arm. In L&D at the free/quality arm, 44.0% (n=11) were properly counseled, and all LARC adopters (n=10) received proper counseling on where and when to have their method removed. Satisfaction with family planning information received ranged from 24.0% (n=6) of L&D clients in the control group to 88.0% (n=22) in the quality arm.

**TABLE 5.** Quality Aspects of Family Planning Method Provision and Counseling, by Department

Outcomes	Arm 1: Quality No. (%)	Arm 2: Free No. (%)	Arm 3: Free/quality No. (%)	Arm 4: Control No. (%)
**Labor and Delivery**	**n=25**	**n=25**	**n=25**	**n=25**
Satisfied with FP information received	22 (88.0)	13 (52.0)	19 (76.0)	6 (24.0)
Modern method provided on day of service	0 (0.0)	0 (0.0)	15 (60.0)	0 (0.0)
Properly counseled on method on day of service	1 (4.0)	0 (0.0)	11 (44.0)	1 (4.0)
LARC provided on day of service	0 (0.0)	0 (0.0)	10 (40.0)	0 (0.0)
IUD provided	0 (0.0)	0 (0.0)	4 (16.0)	0 (0.0)
Implant provided	0 (0.0)	0 (0.0)	6 (24.0)	0 (0.0)
If LARC provided, properly counseled on LARC^a^	NA	NA	10 (100.0)	NA
**Family Planning**	**n=13**	**n=50**	**n=50**	**n=50**
Satisfied with FP information received	13 (100.0)	45 (90.0)	50 (100.0)	45 (90.0)
Modern method provided on day of service	8 (61.5)	41 (82.0)	48 (96.0)	26 (52.0)
Properly counseled on method on day of service	7 (53.9)	32 (64.0)	35 (70.0)	10 (20.0)
LARC provided on day of service	4 (30.8)	30 (60.0)	28 (56.0)	0 (0.0)
IUD provided	3 (23.1)	1 (2.0)	2 (4.0)	0 (0.0)
Implant provided	1 (7.7)	29 (58.0)	26 (52.0)	0 (0.0)
If LARC provided, properly counseled on LARC^a^	4 (100.0)	16 (53.3)	26 (92.9)	0 (0.0)

Abbreviations: FP, family planning; IUD, intrauterine device; LARC, long-acting reversible contraceptives; NA, not applicable.

a Subset of women who received a LARC on the day of service; proper counseling requires that clients were told where the LARC could be removed, when it should be removed due to maximum duration of use, and that it may be removed at any time.

The majority of clients in the family planning unit in each arm reported receiving a modern method on the day of service, ranging from over half (n=26, 52.0%) in the control arm to nearly all (n=48, 96.0%) in the free/quality arm. In the quality arm, the overall sample of family planning clients was very small (n=13) due to low client load; 61.5% (n=8) received a method. In both facilities with free methods, the majority of clients adopted LARCs, while only one-third in the quality arm and no clients in the control arm adopted a LARC. Between 90% and 100% of clients across all study arms reported being satisfied with the family planning information they received (Table 5).

### Method Mix Among Family Planning Services

**FIGURE 3:**
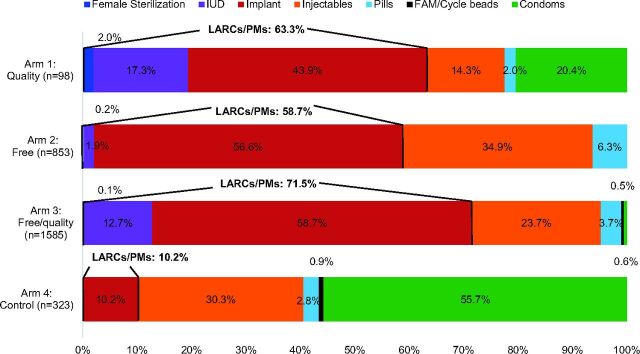
Method Mix Among Family Planning Services Provided During the Study Period, by Study Arm^a,b^ Abbreviations: FAM, fertility awareness method; IUD, intrauterine device; LARCs/PMs, long-acting reversible contraceptives/permanent methods. ^a^Data represent service statistics; therefore, a client who came multiple times for refills of a short-acting method may have been counted more than once. ^b^Data represent 12 months in each study arm.

Analysis of family planning service statistic data indicates that the total number of family planning clients varied dramatically by study arm, with the highest client volume in the free/quality arm (n=1,585), followed by the free arm (n=853), the control group (n=323), and the quality arm (n=97). All intervention arms had significantly higher proportions of clients adopting LARC and permanent methods compared with the control (Quality arm: 63.3%; *P*<.001. Free/quality arm: 58.7%; *P*<.001. Free arm: 71.5%; *P*<.001. Control arm: 10.2%.), and all had lower proportions of clients adopting condoms (Figure 3). The 2 arms with the quality intervention (Arms 1 and 3) had substantial proportions of clients adopting IUDs (17.3% and 12.7%, respectively), while only 1.9% in the free arm and 0 clients in the control arm adopted IUDs. Implants were the most commonly chosen method in all 3 intervention arms (ranging from 43.9% to 58.7%), while condoms were the most common method in the control (55.7%).

### Postpartum Family Planning Reach

The study also analyzed the family planning service statistics to compare the proportions of family planning clients who obtained methods at 0 to 2 days, 3 days to 6 weeks, over 6 weeks to 12 months, and over 12 months postpartum, excluding clients for whom date of last delivery was unknown.

**FIGURE 4:**
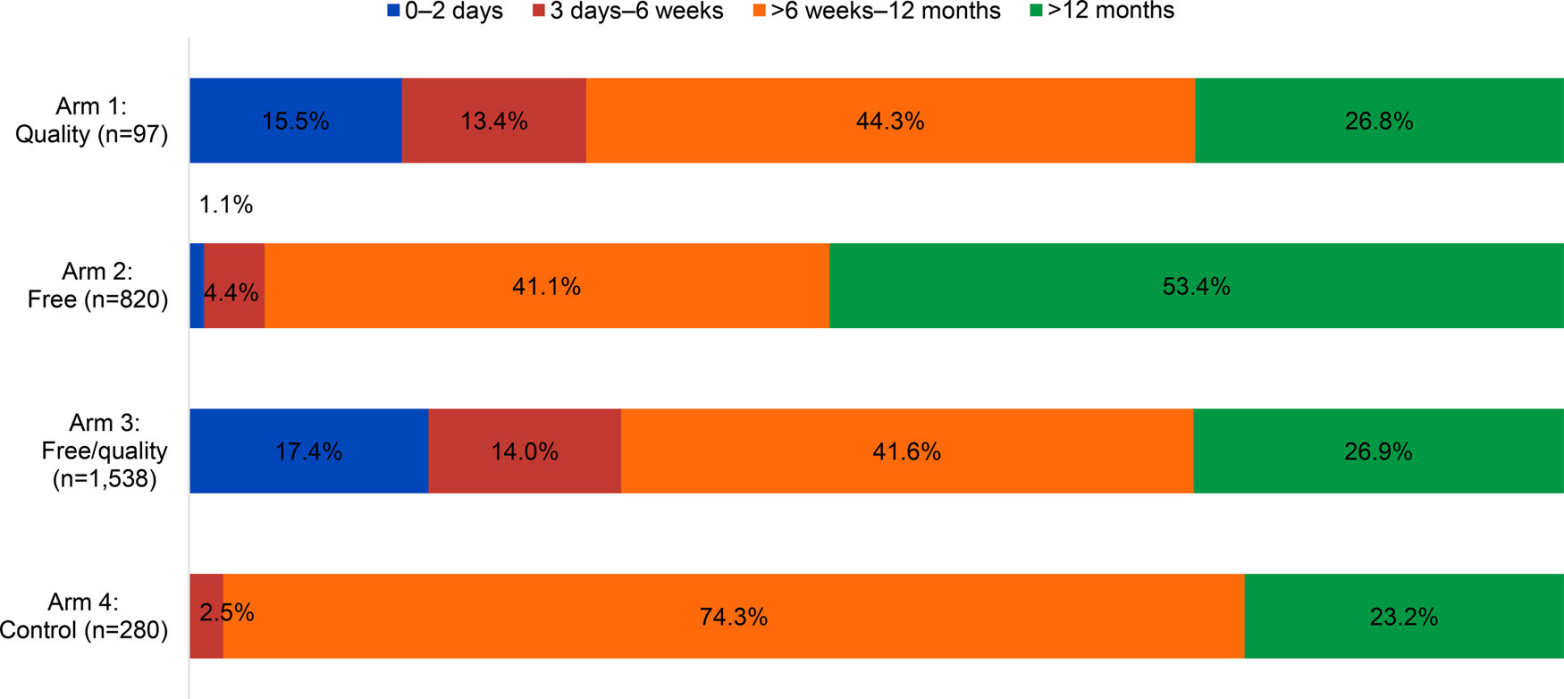
Postpartum Distribution Among Family Planning Clients During the Study Period, by Study Arm^a,b^ ^a^Clients for whom postpartum period was not known were excluded. ^b^Data represent 12 months in each study arm.

Both quality intervention arms had significantly greater proportions of clients adopting methods 0 to 2 days postpartum (Quality arm: 15.5%; *P*<.001. Free/quality arm:17.4%; *P*<.001.) and 3 days to 6 weeks postpartum (Quality arm: 13.4%; *P*<.001. Free/quality arm: 14.0%; *P*<.001.) compared with the controls (0.0% and 2.5%, respectively) (Figure 4). Only the free arm had a significantly different proportion of all family planning clients in the EPP compared with the control group (46.6%; *P*<.001).

The quality intervention arms had higher proportions of client adopting methods 0 to 2 days and 3 days to 6 weeks postpartum than the control group.

### Cost of Methods

In the client interview, women were asked about the influence of cost on their decision to use family planning and on their choice of method. Of the clients who received a method on the day of the interview, more than 65% in 3 of the study arms indicated that the cost of the method choice was somewhat or very important to them (free arm 68.3%, free/quality arm 88.9%, and control 68.0%); in contrast, very few (12.5%) clients in the quality arm said that cost was important (Table 6). Additionally, in the 2 arms with free contraceptives, the majority of clients who received a method were aware that methods were free before coming to the facility (free arm 78.1%, free/quality arm 87.3%). Among women who did not receive a family planning method or who selected an alternate method to her primary method of choice, the method's expense was identified as one of several drivers of method selection in the 2 study arms where methods were not free, although it was not the most common reason cited (Table 6).

**TABLE 6.** Issues Relating to Method Cost Among Women and Reasons for Not Receiving a Method

Outcomes	Arm 1: Quality No. (%)	Arm 2: Free No. (%)	Arm 3: Free/quality No. (%)	Arm 4: Control No. (%)
**Labor and Delivery and Family Planning**	**n=38**	**n=75**	**n=75**	**n=75**
Received a method	8 (21.1)	42 (56.0)	64 (84.0)	25 (33.3)
If received a method, cost was somewhat/very important in method choice	1 (12.5)	28 (68.3)	56 (88.9)	17 (68.0)
If received a method, client was aware method would be free	n/a	32 (78.1)	55 (87.3)	n/a
Did not receive a method/method of choice	30 (79.0)	36 (48.0)	13 (17.3)	60 (80.0)
**Top reasons why client did not receive method/method of choice** ^a^	**n=30**	**n=36**	**n=13**	**n=60**
Wanted to wait 6 weeks/45 days/until period returns or other amount of time	19 (63.3)	18 (50.0)	4 (30.8)	9 (15.0)
Came for a different service	1 (3.3)	5 (13.9)	2 (15.4)	11 (18.3)
No one informed her it was available	1 (3.3)	5 (13.9)	0 (0.0)	9 (15.0)
**Due to expense**	**2 (6.7)**	**0 (0.0)**	**0 (0.0)**	**12 (20.0)**
Decided on a different method	1 (3.3)	1 (2.8)	1 (7.7)	11 (18.3)
Decided not to use	1 (3.3)	3 (8.3)	2 (15.4)	4 (6.7)

a The list of reasons is not exhaustive; clients could give more than 1 answer; the 6 most common responses are listed here.

## DISCUSSION

### Client Characteristics

While women across study groups had many similarities, women at the 2 facilities where methods were not offered for free tended to be wealthier than women in the arms offering free contraception. This could be due to underlying differences in SES in facility catchment areas, or it may indicate self-selection, as poorer women may have purposefully come to the facilities where methods were free. Though no community mobilization was conducted to advertise free services, the majority of family planning clients at these 2 facilities reported that they knew in advance that methods were free, possibly through word of mouth, suggesting they may have come for that reason.

### Screening and Referral

In the study arms that did not include quality inputs (the free and control arms), screening for family planning rarely occurred. This was expected since the screening and referral tool was not introduced to these facilities. We assume any screening that did occur reflects providers asking clients questions unprompted by a tool. Comparatively, the higher incidence of proper screening of clients in the quality arms can be attributed to the introduction of the quality inputs intervention and screening tool. Nevertheless, in both quality facilities, screening overall was still lower than desired (one-third in the free/quality intervention and one-quarter in the quality arm). The goal was universal screening.

While we would not have expected universal referrals, the scarcity of proper referrals being given—only 3 in all of the ANC, PNC, and immunization clients across study arms—indicates this part of the quality intervention was not implemented as designed. While it is true that not all women screened for family planning would have accepted a referral to family planning, the fact that one-third of clients in the free/quality arm were screened but no clients were referred is unlikely to reflect lack of need. Although it is possible that providers gave women paper referrals, they did not keep them; likewise, if oral, not paper, referrals were provided, the women may not have perceived them as such. However, it is more likely that providers simply did not implement this part of the intervention as intended.

Insofar as screening was properly implemented, the greater likelihood of clients being screened at the free/quality arm suggests that the combined cost and quality interventions may have had the greatest effect on provider behavior. However, since neither screening nor referral became universally practiced at any facility, despite frequent follow up with providers throughout the implementation period, this raises questions about the acceptability and feasibility and possibly the method of introduction and supervision of the intervention itself. It is possible that providers at both quality input facilities saw the referral form as an additional burden or they did not have the time to screen and refer each client. If this were determined to be the case, further simplifying the screening and referral forms, or using verbal referrals only, may improve fidelity to this intervention. Further investigation is needed to understand why providers did not fully adopt this practice.

### Family Planning Counseling

Clients in the 2 study groups that offered free contraceptives were significantly more likely to be properly counseled than those in the control group. In contrast, the quality-only study group did not differ from the control. This finding was surprising; we expected both quality input facilities to have improved counseling, with no effect in the free contraceptives-only group. One explanation for this may be the low number of family planning clients in the quality arm. Indeed, when counseling was examined in family planning and L&D settings separately, proper counseling was higher among family planning clients in all 3 study groups and lower among L&D clients, except for in the free/quality study group. We were unable to determine definitively how the quality-only intervention affected counseling, except that providing free contraceptives appeared to positively impact counseling.

Clients offered free contraceptives were significantly more likely to be properly counseled than those in the quality-only and control groups.

### Family Planning Use

In both study arms with free contraceptives, clients were more likely to be modern family planning (excluding condoms), LARC, and implant users, compared with the control group, suggesting that providing free methods may impact family planning use, especially LARC use. More than half of nonpregnant clients in the free/quality arm were using a modern family planning method (excluding condoms), compared with one-fifth in the control arm. Further, one-third of nonpregnant clients in the free/quality arm were using LARCs, compared with only 6% in the control arm. The free/quality arm had the best outcomes in each category of family planning use, supporting our hypothesis that the combined intervention would have the greatest effect. In the quality arm, only the likelihood of being a LARC user was elevated compared with the controls, suggesting that the quality intervention had no impact on family planning use overall. However, interpretation of these results was impeded, as precision and accuracy of the estimates were likely impacted by the small sample size in this group.

### Method Mix

Despite limitations in drawing conclusions for the quality-only study group in particular, examining family planning service data collected over the 12-month intervention period was useful. These data suggest that the intervention had a positive impact on LARC use in all 3 arms, and that the quality intervention, which included the provision of postpartum IUDs, may have increased IUD uptake, even though we could not observe this from the client interviews. However, since the service data represent a full year, and we did not track individual women, users of short-acting methods may have been counted more than once if they returned to get more supplies. This would overrepresent short-acting method users, indicating that the proportion of individual women choosing LARCs may even be higher than calculated here. It is also notable that in the 2 study arms where all methods were free, very few clients chose condoms. The considerable difference in total number of clients in each arm may also indicate preexisting differences between facilities or external factors that affected family planning uptake.

### Postpartum Family Planning Reach

Both study arms with quality inputs had significantly higher proportions of clients served in the immediate postpartum period (0 to 2 days) and within 6 weeks of delivery, compared with the control arm. These results are consistent with the expected results of the quality intervention, which aimed to introduce access to family planning in the L&D ward (0 to 2 days) and to update providers on the increasing array of methods available to women within 6 weeks of delivery. Specifically, the MEC update and postpartum IUD training made implants and IUDs available to women immediately postpartum in the 2 quality intervention arms (Arms 1 and 3). In contrast, in the facilities without quality inputs (the free arm and control arm), no methods were available in the L&D ward and only condoms and progestin-only oral contraceptives were available within 6 weeks postpartum in the family planning unit. Accordingly, we observed very few clients obtaining methods within 6 weeks postpartum in the free or control arms. This suggests that the quality intervention may have influenced when women adopt family planning and increased postpartum family planning use. It is likely that the availability of free family planning services in Arms 1 and 2 affected the number of family planning clients attending these facilities; however pre-existing and external factors likely also affected client load.

### Cost of Methods

Most family planning clients in the free, free/quality, and control arms indicated that cost was somewhat or very important in their method choice. Conversely, only a few women who did not receive a method or did not receive their method of choice stated that expense was the main reason. This could be interpreted to mean that cost is only an important barrier for a small portion of the population, or it could reflect a self-selecting sample (i.e., women for whom cost is a barrier would not have come to facilities where they had to pay for a method, and thus would not have been interviewed). The cost of methods normally varies among facilities, outside the context of the study; as a result, we were unable to measure to what extent this affected if and where women sought contraceptive services. Interpreted in light of other study findings, specifically, that free facilities had higher modern family planning (excluding condoms) and LARC use, cost as a barrier seems likely and suggests that being able to obtain methods for free may be a key factor to increase family planning use in Kinshasa. However, given external factors may also affect client flow, and that baseline data are not available, we cannot conclude whether this relationship is causal.

### Limitations

The study design used to evaluate the interventions was selected primarily for its feasibility in the given context and circumstances, with some expense to rigor. The lack of pretest data and randomization, coupled with having only 1 facility per study group, limited the validity and generalizability of our findings. Facility-level characteristics varied in terms of the number of providers trained to provide family planning, client load, and client demographics, among other known and unknown factors. These differences may well have affected implementation of the study by providers as well as client-level outcomes. While we controlled for client characteristics that varied between intervention and control facilities, this was insufficient to conclude that the study facilities were equivalent to each other analytically and to attribute differences in intervention performance entirely to the interventions themselves. Two of the sites, the free arm and the free/quality arm, received support from ExpandFP for 2 years prior to the study launch, which included the clinical training of providers and monthly special family planning days with free contraception. Any impact of the earlier support on the study is believed to be minimal, given that the support activities did not have screening, referral, integration, or PPFP elements, and that services were only free during distinct events. However, it is possible that these facilities were more well-known in the community for providing family planning, specifically LARCs, and this may have contributed to high client load in these facilities. A dispute over the land on which 1 of the facilities is built (the quality arm) arose during implementation, which we believe affected overall client flow, resulting in a lower-than-desired sample in this group, as well as provider morale or performance. This situation has implications for the interpretation of data in this particular group.

It is possible that some women were interviewed more than once, on different dates. We mentioned earlier that women using short-acting methods may have been counted more than once; this may also have been the case if a woman was interviewed in L&D after giving birth and then again in PNC 2 weeks later. However, being interviewed more than once should not have altered a woman's reporting of her experience in a particular service, and any risk to measures of family planning use was likely small and distributed equivalently across study groups.

Service statistics were compared across groups as an average measure; as a result, this analysis does not fully account for changes over time that may have occurred at different rates in each study group. Finally, client exit interviews were conducted when facilities had been implementing the intervention for between 9 and 12 months. It is possible that the different time periods affected levels of intervention uptake, though monitoring of referrals throughout the intervention period did not indicate substantial changes over time. Finally, the relatively short period of implementation limited conclusions on the sustainability of such an intervention.

## CONCLUSIONS

While the results of this study are limited, they do have important implications for programs operating in this and other low-resource settings where use of family planning, especially family planning within the EPP, is limited. The results provide some preliminary evidence of how integrating a PPFP intervention across service delivery units within a facility may improve family planning uptake in the EPP, and how different types of interventions (cost and quality improvements) can work together to compound improvements in PPFP uptake in highly difficult programmatic contexts.

In light of this, we conclude that combining the free contraceptives and quality interventions had the strongest effect on family planning screening, quality of counseling, modern family planning use, and LARCs use, specifically. Further, family planning clients in the 2 quality intervention arms were more likely to adopt a method within 6 weeks of delivery, indicating that this intervention better targets postpartum women than offering free contraceptives alone. The quality-only intervention performed well on improving screening practices but not on most other indicators, suggesting that quality interventions may be necessary but insufficient to effect change. Still, this is not conclusive, considering problems with sample size already discussed. As expected, the cost-only intervention did not improve screening or referral but was associated with improved counseling practices and modern family planning use overall.

Combining free contraceptives with quality services appears to be more effective than implementing each type of intervention alone.

Providing clients with access to free contraceptives is key to improving family planning use in this setting and warrants further investigation into how to make free services available and financially sustainable for the health system. Training providers to properly counsel, screen, and refer clients and to provide LARCs and other methods in the postpartum period is also crucial to improving family planning access and use. Future scale up of this or similar interventions should investigate how to adjust screening and referral practices so that they can be implemented more fully and consistently. Combining these interventions appears to be more effective than implementing either intervention alone, and addressing multiple barriers to family planning use simultaneously is necessary to effect meaningful change in access to PPFP.

## Notes

**En français**

**Interventions sur la qualité et le coût pendant la période périnatale prolongée pour accroître l'utilisation de la planification familiale à Kinshasa, RDC : Résultats d'une étude initiale**

L'intervention combinée reposant sur les contraceptifs gratuits et un ensemble d'apports de qualité pour la planification familiale lors de la période périnatale prolongée, notamment la mise à disposition de méthodes de longue durée d'action immédiatement après l'accouchement, ont eu un impact considérable sur l'utilisation de contraceptifs modernes, en particulier les méthodes de longue durée d'action.

**RÉSUMÉ**

**Contexte :** À travers le monde, la plupart des femmes n'envisagent pas une autre grossesse dans l'année qui suit un accouchement, mais l'adoption d'une contraception moderne pendant cette période reste faible. Nous avons testé de manière indépendante, 2 approches de l'augmentation de l'adoption de la contraception et les 2 approches combinées en s'appuyant sur une étude quasi-expérimentale à Kinshasa, en République démocratique du Congo.

**Méthodes :** Les données analytiques primaires proviennent des entretiens menés auprès des clientes après l'intervention (N=563) issues de 4 groupes d'étude. Le premier groupe (n=150) a bénéficié d'une planification familiale gratuite, et le second groupe (n=113) a bénéficié d'une intervention fondée sur les apports de qualité impliquant le dépistage systématique, l'orientation vers d'autres structures et la mise à disposition immédiate de contraceptifs réversibles de longue durée d'action après le travail et l'accouchement. Le troisième groupe (n=150) a bénéficié de 2 interventions et aucune intervention n'a été consacrée au quatrième groupe (n=150). Des statistiques issues des services de planification familiale ont également été collectées pendant la période d'intervention.

**Résultats :** Les femmes du groupe de qualité (coefficients de probabilité [CP]=4,5; 95% d'intervalle de confiance [IC], 1,8 à 10,9) et du groupe gratuit/qualité (CP=6,7; 95% d'IC, de 2,8 à 16,1) avaient plus de chances de subir un test dépistage correct que les femmes du groupe de contrôle, mais l'orientation vers d'autres structures par le biais de supports papier a rarement été mise en œuvre dans un des groupes. Les femmes du groupe libre (CP=3,8; 95% d'IC, 1,6 à 9,0) et dans le groupe gratuit/qualité (CP=11,0; 95% d'IC, 4,3 à 27,9) étaient plus susceptibles que le groupe de contrôle, de déclarer qu'elles avaient été conseillées correctement sur la planification familiale. Les clientes étaient plus susceptibles d'être des utilisatrices de contraception moderne (à l'exclusion des préservatifs) dans le groupe gratuit (CP=3,2; 95% d'IC, de 1,4 à 7,2) et dans le groupe gratuit/qualité (CP=8,6; 95 IC, de 3,9 à 19,0) que dans le groupe de contrôle. Les clientes de l'ensemble des groupes d'étude étaient plus susceptibles d'utiliser une méthode réversible de longue durée d'action en comparaison avec le groupe de contrôle (Groupe qualité : CP=2,9; 95% d'IC, de 1,1 à 7,9. Groupe gratuit : CP=5,6; 95% d'IC, 2,3 à 13,7. Groupe gratuit/qualité : CP=8,4; 95% d'IC, 3,4 à 20,6). Les statistiques de service issues du groupe d'intervention combinée ont indiqué qu'une proportion plus élevée de l'adoption de la planification familiale a eu lieu pendant la période du postpartum immédiat (0 à 2 jours) dans le groupe qualité (*P*<,001) et le groupe gratuit/qualité (*P*<,001) par rapport au groupe de contrôle. Les apports de qualité, les contraceptifs gratuits, et l'intervention combinée ont eu des impacts positifs sur certains aspects du dépistage et de l'adoption de contraceptifs. L'intervention combinée a obtenu les meilleurs résultats, à tout point de vue.

**Conclusion :** Dans ce contexte, la mise à disposition de la planification familiale y compris des méthodes réversibles de longue durée d'action pendant la période du postpartum immédiat, un système de dépistage et d'orientation vers d'autres structures, ainsi que l'offre de méthodes gratuites peuvent améliorer l'accès et l'adoption de la planification familiale durant la période périnatale prolongée.

## Notes

**Peer Reviewed**

**Funding:** Bill & Melinda Gates Foundation.
